# Crystal structure of 1-(1-methyl-1*H*-imidazol-2-yl)-4-phenyl-1*H*-1,2,3-triazole dihydrate

**DOI:** 10.1107/S2056989015020721

**Published:** 2015-11-14

**Authors:** Simone Haslinger, Gerhard Laus, Klaus Wurst, Herwig Schottenberger

**Affiliations:** a University of Innsbruck, Faculty of Chemistry and Pharmacy, Innrain 80, 6020 Innsbruck, Austria

**Keywords:** crystal structure, 1*H*-imidazole, 1,2,3-triazole, hydrate, hydrogen bonding

## Abstract

The title compound, C_12_H_11_N_5_·2H_2_O, which crystallizes as a dihydrate, was obtained by Cu^I^-catalysed azide–alkyne cyclo­addition from 2-azido-1-methyl­imidazole and phenyl­ethyne. The dihedral angles between the central triazole ring (r.m.s. deviation = 0.004 Å) and the pendant imidazole (r.m.s. deviation = 0.006 Å) and phenyl rings are 12.3 (2) and 2.54 (19)°, respectively. In the crystal, the water mol­ecules are connected into [010] chains by O—H⋯O hydrogen bonds, while O—H⋯N hydrogen bonds connect the water mol­ecules to the organic mol­ecules, generating corrugated (100) sheets.

## Related literature   

For the synthesis and thermal cyclo­addition of 2-azido-1-methyl­imidazole, see: Zanirato & Cerini (2005 ▸). For related structures, see: Ramana & Punniyamurthy (2012 ▸). For background to 1,2,3-triazoles as peptidomimetics, see: Angell & Burgess (2007 ▸); Pedersen & Abell (2011 ▸); Tron *et al.* (2008 ▸). For copper(I)-catalysed azide–alkyne cyclo­additions, see: Haldón *et al.* (2015 ▸); Meldal & Tornoe (2008 ▸); Rostovtsev *et al.* (2002 ▸).
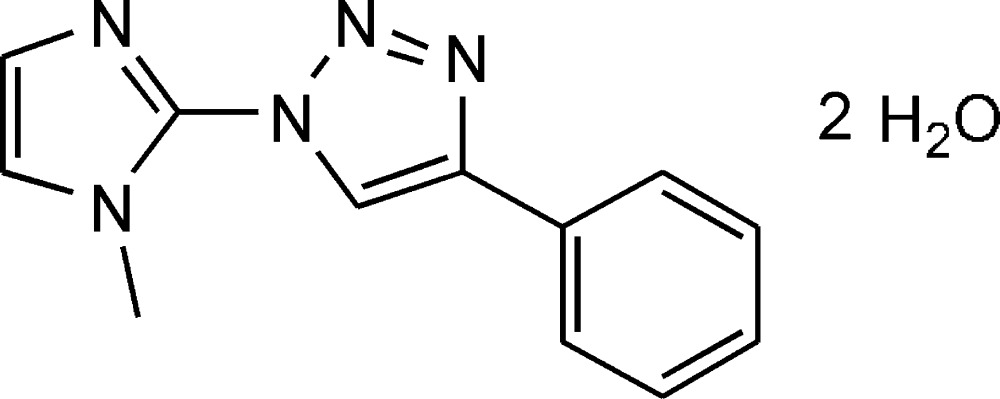


## Experimental   

### Crystal data  

C_12_H_11_N_5_·2H_2_O
*M* *_r_* = 261.29
Orthorhombic, 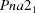

*a* = 18.8585 (9) Å
*b* = 4.7884 (2) Å
*c* = 14.4285 (6) Å
*V* = 1302.92 (10) Å^3^
*Z* = 4
Mo *K*α radiation
μ = 0.10 mm^−1^
*T* = 233 K
0.40 × 0.05 × 0.05 mm

### Data collection  

Nonius KappaCCD diffractometer
6624 measured reflections
2277 independent reflections
1878 reflections with *I* > 2σ(*I*)
*R*_int_ = 0.050

### Refinement  

*R*[*F* ^2^ > 2σ(*F* ^2^)] = 0.045
*wR*(*F* ^2^) = 0.088
*S* = 1.08
2277 reflections
190 parameters
5 restraints
H atoms treated by a mixture of independent and constrained refinement
Δρ_max_ = 0.15 e Å^−3^
Δρ_min_ = −0.15 e Å^−3^

### 

Data collection: *COLLECT* (Hooft, 1998 ▸); cell refinement: *DENZO* and *SCALEPACK* (Otwinowski & Minor, 1997 ▸); data reduction: *DENZO* and *SCALEPACK*; program(s) used to solve structure: *SHELXTL* (Sheldrick, 2008 ▸); program(s) used to refine structure: *SHELXL2014* (Sheldrick, 2015 ▸); molecular graphics: *ORTEP-3 for Windows* (Farrugia, 2012 ▸) and *Mercury* (Macrae *et al.*, 2006 ▸); software used to prepare material for publication: *SHELXL2014*.

## Supplementary Material

Crystal structure: contains datablock(s) I. DOI: 10.1107/S2056989015020721/hb7534sup1.cif

Structure factors: contains datablock(s) I. DOI: 10.1107/S2056989015020721/hb7534Isup2.hkl

Supporting information file. DOI: 10.1107/S2056989015020721/hb7534Isup3.mol

Supporting information file. DOI: 10.1107/S2056989015020721/hb7534Isup4.cml

**Fig. 1.**
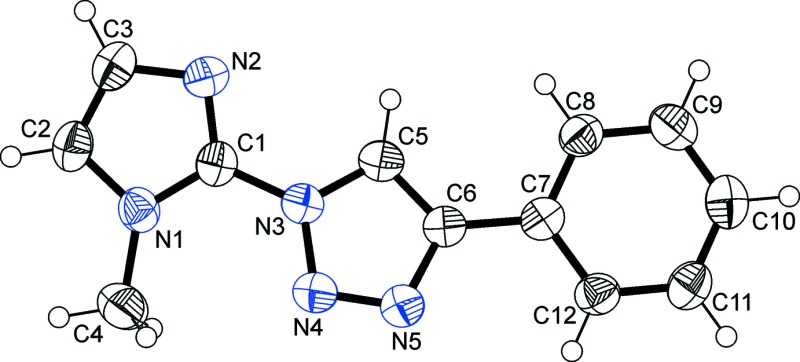
The molecular structure of the title compound, with atom labels and 50% probability displacement ellipsoids for non-H atoms. The water molecules are not shown.

. DOI: 10.1107/S2056989015020721/hb7534fig1.tif
The mol­ecular structure of the title compound, with atom labels and 50% probability displacement ellipsoids for non-H atoms. The water mol­ecules are not shown.

**Fig. 2.**
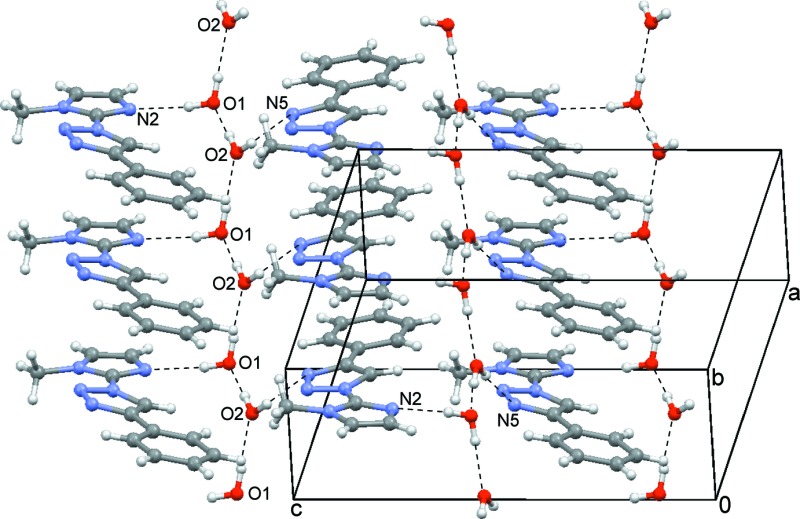
Infinite chains of hydrogen-bonded water molecules link the heterocyclic molecules.

. DOI: 10.1107/S2056989015020721/hb7534fig2.tif
Infinite chains of hydrogen-bonded water mol­ecules link the heterocyclic mol­ecules.

CCDC reference: 1434671

Additional supporting information:  crystallographic information; 3D view; checkCIF report

## Figures and Tables

**Table 1:** Hydrogen-bond geometry (Å, °)

*D*—H⋯*A*	*D*—H	H⋯*A*	*D*⋯*A*	*D*—H⋯*A*
O1—H1*A*⋯N2	0.85 (3)	2.02 (3)	2.863 (4)	172 (4)
O1—H1*B*⋯O2^i^	0.86 (3)	1.91 (4)	2.768 (4)	176 (4)
O2—H2*A*⋯N5^ii^	0.82 (4)	2.19 (4)	3.007 (4)	174 (4)
O2—H2*B*⋯O1	0.84 (4)	1.92 (4)	2.750 (4)	170 (4)

Symmetry codes: (i) 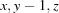; (ii) 
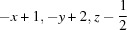
.

## supplementary crystallographic information

### S1. Comment

Due to the structural and stereoelectronic similarity between 1,2,3-triazoles and amide bonds, this class of heterocycles holds promising potential as peptidomimetics (Angell & Burgess, 2007; Pedersen & Abell, 2011; Tron *et al.*, 2008) with improved biological activities. The molecular structure of the title compound is shown in Figure 1. The interplanar angle between the imidazole and triazole rings is 12.3°, whereas the angle between the triazole and phenyl ring planes is only 2.5°. The two independent water molecules form infinite chains *via* O—H···O hydrogen bonds and donate O1—H···N2 and O2—H···N5 hydrogen bonds to the imidazole and triazole moieties (Figure 2). The hydrogen bond geometries are summarized in Table 1.

### S2. Experimental

The thermal cycloaddition of azidoazoles with (trimethylsilyl)ethyne has been reported to require extraordinarily long reaction times and chromatographic separation of the resulting isomers (Zanirato & Cerini, 2005). On the other hand, copper(I)-catalysed azide-alkyne cycloadditions (Haldón *et al.*, 2015; Meldal & Tornoe, 2008; Rostovtsev *et al.*, 2002) exhibit more favorable rates and excellent selectivities. The 2-azido-1-methylimidazole was prepared by lithiation and azidation of 1-methylimidazole followed by fragmentation of the resulting tosyltriazenyl salt according to the literature (Zanirato & Cerini, 2005). Thus, the aqueous solution (20 ml) of 2-azido-1-methylimidazole (1.1 mmol) was deoxygenated in a stream of argon. Phenylethyne (0.14 ml, 1.2 mmol), CuSO_4_^.^5H_2_O (27 mg, 0.1 mmol) and sodium ascorbate (64 mg, 0.3 mmol) were subsequently added. This suspension was stirred at room temperature for 20 h. The white precipitate was collected by filtration and recrystallized from a mixture of acetone (10 ml) and H_2_O (0.5 ml) to yield colorless needles (47 mg, 20%). Melting point: 77 °C. IR (neat): ν 1546, 1514, 1478, 1278, 1024, 1005, 762, 689 cm^-1. 1^H NMR (DMSO-d_6_, 300 MHz): δ 3.72(s, 3H), 7.06 (s, 1H), 7.39 (t, 1H; *J* = 7.6 Hz), 7.42 (s, 1H), 7.50 (t, 2H; *J* = 7.6 Hz), 7.99 (d, 2H; *J* = 7.6 Hz), 9.13 (s, 1H) p.p.m. ^13^C NMR (DMSO-d_6_, 75 MHz): δ 33.5, 122.8, 123.3, 125.5 (2 C), 126.3, 128.5, 129.0 (2 C), 129.8, 136.9, 146.4 p.p.m.

### S3. Refinement

Carbon-bound H atoms were placed in calculated positions and refined riding on their respective carbon atom. Methyl H atoms were fitted to the experimental electron density by allowing them to rotate around the C—C bond with a fixed angle (HFIX 137). Isotropic displacement parameters were constrained with *U*_iso_(H) = 1.5*U*_eq_(C) for methyl H atoms and 1.2*U*_eq_(C) for other H atoms. The H atoms of the water molecules were located from a Fourier map and restrained with a distance of O—H = 0.83 (2) Å.

### Crystal data

**Table:**

C_12_H_11_N_5_·2H_2_O	*D*_x_ = 1.332 Mg m^−^^3^
*M**_r_* = 261.29	Mo *K*α radiation, λ = 0.71073 Å
Orthorhombic, *P**n**a*2_1_	Cell parameters from 18840 reflections
*a* = 18.8585 (9) Å	θ = 1.0–25.2°
*b* = 4.7884 (2) Å	µ = 0.10 mm^−^^1^
*c* = 14.4285 (6) Å	*T* = 233 K
*V* = 1302.92 (10) Å^3^	Prism, colourless
*Z* = 4	0.40 × 0.05 × 0.05 mm
*F*(000) = 552	

### Data collection

**Table:**

Nonius KappaCCD diffractometer	*R*_int_ = 0.050
Detector resolution: 9.4 pixels mm^-1^	θ_max_ = 25.0°, θ_min_ = 2.2°
phi– and ω–scans	*h* = −18→22
6624 measured reflections	*k* = −5→5
2277 independent reflections	*l* = −17→17
1878 reflections with *I* > 2σ(*I*)	

### Refinement

**Table:**

Refinement on *F*^2^	Hydrogen site location: mixed
Least-squares matrix: full	H atoms treated by a mixture of independent and constrained refinement
*R*[*F*^2^ > 2σ(*F*^2^)] = 0.045	*w* = 1/[σ^2^(*F*_o_^2^) + (0.0315*P*)^2^ + 0.2291*P*] where *P* = (*F*_o_^2^ + 2*F*_c_^2^)/3
*wR*(*F*^2^) = 0.088	(Δ/σ)_max_ < 0.001
*S* = 1.08	Δρ_max_ = 0.15 e Å^−^^3^
2277 reflections	Δρ_min_ = −0.15 e Å^−^^3^
190 parameters	Extinction correction: *SHELXL2014* (Sheldrick 2015), Fc^*^=kFc[1+0.001xFc^2^λ^3^/sin(2θ)]^-1/4^
5 restraints	Extinction coefficient: 0.018 (3)

### Special details

**Table:**

Geometry. All e.s.d.'s (except the e.s.d. in the dihedral angle between two l.s. planes) are estimated using the full covariance matrix. The cell e.s.d.'s are taken into account individually in the estimation of e.s.d.'s in distances, angles and torsion angles; correlations between e.s.d.'s in cell parameters are only used when they are defined by crystal symmetry. An approximate (isotropic) treatment of cell e.s.d.'s is used for estimating e.s.d.'s involving l.s. planes.
Refinement. Hydrogen atoms at water molecules were found and refined isotropically with a bond restraint, d = 0.83 (2) Å.

### Fractional atomic coordinates and isotropic or equivalent isotropic displacement parameters (Å^2^)

**Table:**

	*x*	*y*	*z*	*U*_iso_*/*U*_eq_	
N1	0.71035 (16)	0.4793 (6)	1.0472 (2)	0.0432 (7)	
N2	0.69605 (16)	0.5200 (6)	0.89493 (19)	0.0439 (7)	
N3	0.62108 (14)	0.8035 (6)	0.98838 (18)	0.0384 (7)	
N4	0.58989 (17)	0.8522 (7)	1.0712 (2)	0.0504 (8)	
N5	0.54108 (16)	1.0443 (6)	1.0565 (2)	0.0491 (8)	
C1	0.67522 (16)	0.6024 (7)	0.9768 (3)	0.0387 (8)	
C2	0.75758 (19)	0.2996 (8)	1.0060 (3)	0.0484 (9)	
H2	0.7900	0.1815	1.0361	0.058*	
C3	0.74817 (19)	0.3260 (7)	0.9139 (3)	0.0487 (9)	
H3	0.7736	0.2260	0.8687	0.058*	
C4	0.7052 (2)	0.5242 (10)	1.1475 (3)	0.0698 (13)	
H4A	0.6585	0.4685	1.1688	0.105*	
H4B	0.7127	0.7203	1.1612	0.105*	
H4C	0.7409	0.4133	1.1788	0.105*	
C5	0.59235 (19)	0.9645 (7)	0.9213 (3)	0.0404 (9)	
H5	0.6047	0.9694	0.8582	0.048*	
C6	0.54150 (17)	1.1183 (7)	0.9651 (2)	0.0368 (8)	
C7	0.49322 (18)	1.3296 (7)	0.9261 (2)	0.0387 (8)	
C8	0.49478 (18)	1.3869 (8)	0.8321 (2)	0.0446 (9)	
H8	0.5263	1.2892	0.7935	0.054*	
C9	0.4501 (2)	1.5875 (8)	0.7944 (3)	0.0523 (10)	
H9	0.4516	1.6260	0.7306	0.063*	
C10	0.4035 (2)	1.7299 (8)	0.8506 (3)	0.0525 (10)	
H10	0.3732	1.8651	0.8250	0.063*	
C11	0.4012 (2)	1.6750 (8)	0.9445 (3)	0.0492 (10)	
H11	0.3690	1.7716	0.9826	0.059*	
C12	0.44632 (18)	1.4772 (6)	0.9824 (3)	0.0442 (9)	
H12	0.4453	1.4423	1.0465	0.053*	
O1	0.66022 (15)	0.5304 (6)	0.70215 (18)	0.0527 (8)	
O2	0.59225 (17)	1.0282 (6)	0.6683 (2)	0.0566 (8)	
H1A	0.675 (3)	0.533 (11)	0.758 (2)	0.12 (2)*	
H1B	0.641 (2)	0.371 (7)	0.692 (3)	0.079 (15)*	
H2A	0.5546 (17)	1.019 (9)	0.640 (3)	0.077 (16)*	
H2B	0.608 (2)	0.869 (7)	0.680 (4)	0.081 (16)*	

### Atomic displacement parameters (Å^2^)

**Table:**

	*U*^11^	*U*^22^	*U*^33^	*U*^12^	*U*^13^	*U*^23^
N1	0.0357 (16)	0.0487 (17)	0.0453 (18)	−0.0013 (15)	−0.0035 (14)	0.0036 (14)
N2	0.0433 (17)	0.0464 (17)	0.0419 (18)	−0.0012 (14)	0.0035 (15)	−0.0052 (13)
N3	0.0364 (15)	0.0410 (16)	0.0377 (16)	−0.0046 (13)	0.0002 (15)	−0.0002 (14)
N4	0.0510 (18)	0.0620 (19)	0.0384 (17)	0.0082 (17)	0.0022 (15)	0.0006 (15)
N5	0.0471 (18)	0.057 (2)	0.0431 (17)	0.0070 (16)	0.0016 (15)	−0.0006 (15)
C1	0.0302 (17)	0.0374 (18)	0.048 (2)	−0.0061 (15)	0.0008 (16)	0.0014 (17)
C2	0.0373 (19)	0.047 (2)	0.061 (2)	0.0046 (18)	0.0009 (19)	0.0036 (19)
C3	0.040 (2)	0.045 (2)	0.061 (3)	0.0021 (17)	0.0027 (19)	−0.005 (2)
C4	0.059 (3)	0.105 (4)	0.045 (3)	0.014 (3)	−0.003 (2)	0.006 (2)
C5	0.042 (2)	0.0401 (19)	0.0391 (19)	−0.0072 (17)	0.0023 (17)	0.0005 (17)
C6	0.0364 (18)	0.0347 (18)	0.0394 (18)	−0.0070 (15)	0.0018 (16)	−0.0004 (16)
C7	0.0355 (19)	0.0357 (19)	0.0449 (19)	−0.0075 (16)	0.0023 (16)	−0.0023 (17)
C8	0.040 (2)	0.048 (2)	0.045 (2)	0.0015 (18)	0.0019 (18)	−0.0053 (17)
C9	0.050 (2)	0.062 (3)	0.044 (2)	−0.005 (2)	−0.0038 (19)	0.006 (2)
C10	0.049 (2)	0.045 (2)	0.064 (3)	0.0005 (19)	−0.008 (2)	0.004 (2)
C11	0.048 (2)	0.041 (2)	0.059 (3)	0.0023 (19)	0.0046 (17)	−0.0021 (17)
C12	0.050 (2)	0.040 (2)	0.043 (2)	−0.0013 (17)	0.0071 (18)	0.0018 (17)
O1	0.0603 (19)	0.0462 (17)	0.0514 (19)	−0.0029 (14)	−0.0028 (14)	0.0066 (13)
O2	0.062 (2)	0.0472 (17)	0.0608 (18)	0.0040 (15)	−0.0141 (16)	−0.0078 (14)

### Geometric parameters (Å, º)

**Table:**

N1—C1	1.347 (5)	C5—H5	0.9400
N1—C2	1.374 (4)	C6—C7	1.472 (5)
N1—C4	1.467 (5)	C7—C8	1.385 (5)
N2—C1	1.306 (5)	C7—C12	1.393 (5)
N2—C3	1.380 (5)	C8—C9	1.389 (5)
N3—C5	1.350 (4)	C8—H8	0.9400
N3—N4	1.352 (4)	C9—C10	1.376 (5)
N3—C1	1.413 (4)	C9—H9	0.9400
N4—N5	1.318 (4)	C10—C11	1.380 (5)
N5—C6	1.366 (5)	C10—H10	0.9400
C2—C3	1.347 (5)	C11—C12	1.386 (5)
C2—H2	0.9400	C11—H11	0.9400
C3—H3	0.9400	C12—H12	0.9400
C4—H4A	0.9700	O1—H1A	0.85 (3)
C4—H4B	0.9700	O1—H1B	0.86 (3)
C4—H4C	0.9700	O2—H2A	0.82 (3)
C5—C6	1.364 (5)	O2—H2B	0.84 (3)
			
C1—N1—C2	105.5 (3)	N3—C5—H5	127.5
C1—N1—C4	130.3 (3)	C6—C5—H5	127.5
C2—N1—C4	124.2 (3)	C5—C6—N5	108.1 (3)
C1—N2—C3	103.8 (3)	C5—C6—C7	129.0 (3)
C5—N3—N4	111.1 (3)	N5—C6—C7	122.9 (3)
C5—N3—C1	126.5 (3)	C8—C7—C12	118.9 (3)
N4—N3—C1	122.4 (3)	C8—C7—C6	119.8 (3)
N5—N4—N3	106.4 (3)	C12—C7—C6	121.3 (3)
N4—N5—C6	109.4 (3)	C7—C8—C9	120.5 (3)
N2—C1—N1	113.7 (3)	C7—C8—H8	119.7
N2—C1—N3	122.0 (3)	C9—C8—H8	119.7
N1—C1—N3	124.4 (3)	C10—C9—C8	120.0 (4)
C3—C2—N1	106.4 (3)	C10—C9—H9	120.0
C3—C2—H2	126.8	C8—C9—H9	120.0
N1—C2—H2	126.8	C9—C10—C11	120.3 (4)
C2—C3—N2	110.6 (3)	C9—C10—H10	119.9
C2—C3—H3	124.7	C11—C10—H10	119.9
N2—C3—H3	124.7	C10—C11—C12	119.8 (4)
N1—C4—H4A	109.5	C10—C11—H11	120.1
N1—C4—H4B	109.5	C12—C11—H11	120.1
H4A—C4—H4B	109.5	C11—C12—C7	120.5 (3)
N1—C4—H4C	109.5	C11—C12—H12	119.8
H4A—C4—H4C	109.5	C7—C12—H12	119.8
H4B—C4—H4C	109.5	H1A—O1—H1B	108 (5)
N3—C5—C6	105.0 (3)	H2A—O2—H2B	111 (5)
			
C5—N3—N4—N5	0.2 (4)	C1—N3—C5—C6	178.4 (3)
C1—N3—N4—N5	−178.3 (3)	N3—C5—C6—N5	−0.3 (4)
N3—N4—N5—C6	−0.4 (4)	N3—C5—C6—C7	180.0 (3)
C3—N2—C1—N1	0.8 (4)	N4—N5—C6—C5	0.4 (4)
C3—N2—C1—N3	−179.6 (3)	N4—N5—C6—C7	−179.8 (3)
C2—N1—C1—N2	−0.6 (4)	C5—C6—C7—C8	2.1 (5)
C4—N1—C1—N2	176.7 (4)	N5—C6—C7—C8	−177.6 (3)
C2—N1—C1—N3	179.8 (3)	C5—C6—C7—C12	−177.0 (3)
C4—N1—C1—N3	−2.9 (6)	N5—C6—C7—C12	3.3 (5)
C5—N3—C1—N2	−10.9 (5)	C12—C7—C8—C9	−0.2 (5)
N4—N3—C1—N2	167.3 (3)	C6—C7—C8—C9	−179.4 (3)
C5—N3—C1—N1	168.6 (3)	C7—C8—C9—C10	−0.4 (5)
N4—N3—C1—N1	−13.2 (5)	C8—C9—C10—C11	0.2 (6)
C1—N1—C2—C3	0.2 (4)	C9—C10—C11—C12	0.5 (6)
C4—N1—C2—C3	−177.4 (4)	C10—C11—C12—C7	−1.1 (5)
N1—C2—C3—N2	0.3 (4)	C8—C7—C12—C11	1.0 (5)
C1—N2—C3—C2	−0.6 (4)	C6—C7—C12—C11	−179.9 (3)
N4—N3—C5—C6	0.1 (4)		

### Hydrogen-bond geometry (Å, º)

**Table:**

*D*—H···*A*	*D*—H	H···*A*	*D*···*A*	*D*—H···*A*
O1—H1*A*···N2	0.85 (3)	2.02 (3)	2.863 (4)	172 (4)
O1—H1*B*···O2^i^	0.86 (3)	1.91 (4)	2.768 (4)	176 (4)
O2—H2*A*···N5^ii^	0.82 (4)	2.19 (4)	3.007 (4)	174 (4)
O2—H2*B*···O1	0.84 (4)	1.92 (4)	2.750 (4)	170 (4)

Symmetry codes: (i) *x*, *y*−1, *z*; (ii) −*x*+1, −*y*+2, *z*−1/2.
