# Supplementary figures and images for: Crystal structure of 1-(1-methyl-1H-imidazol-2-yl)-4-phenyl-1H-1,2,3-triazole dihydrate
Source: Acta Crystallogr E Crystallogr Commun. 2015 Nov 14;71(Pt 12):o945–6. doi: 10.1107/S2056989015020721 (PMC4719915; doi:10.1107/S2056989015020721)

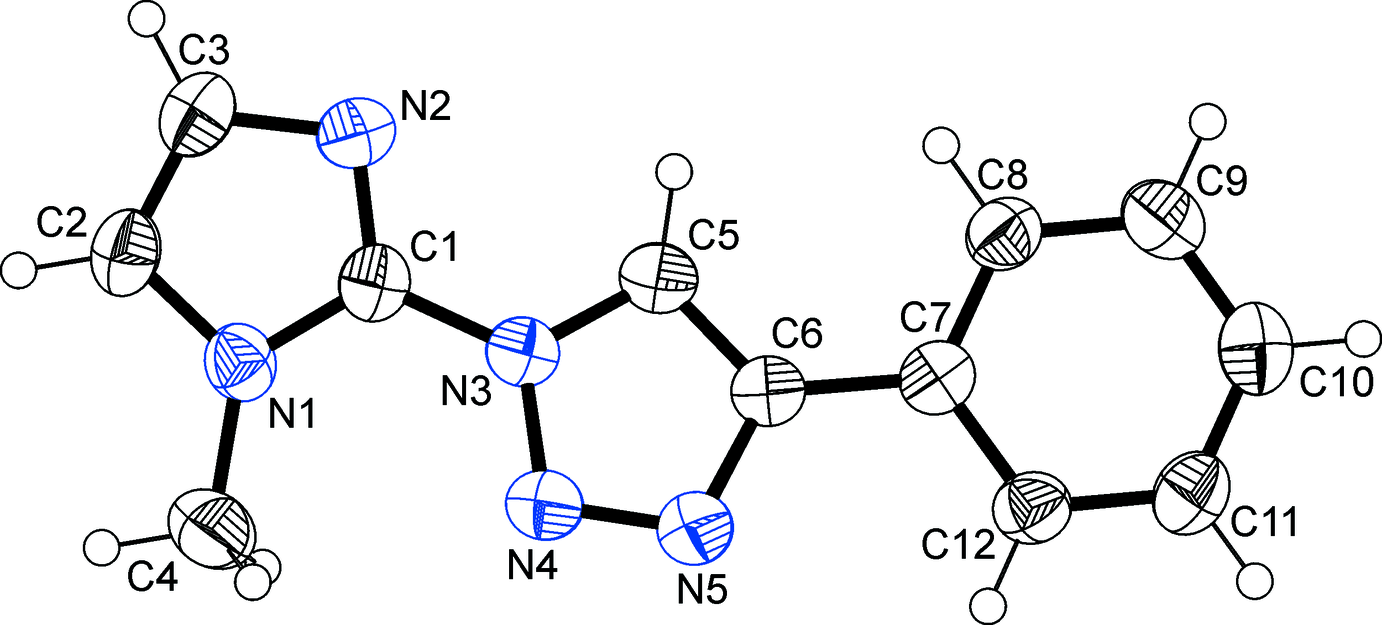

Supplement: Supplementary file 5 [file e-71-0o945-fig1.tif]

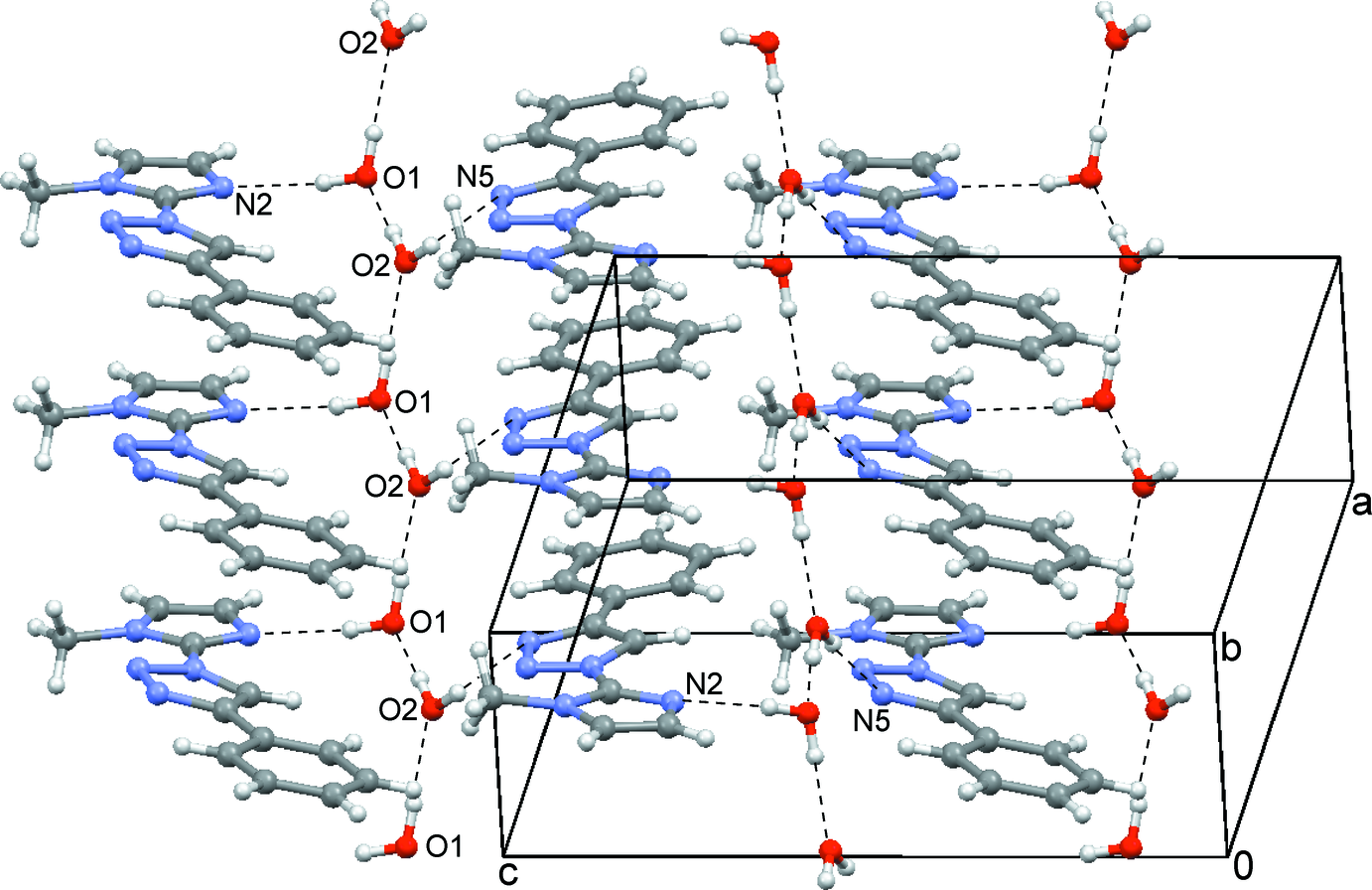

Supplement: Supplementary file 6 [file e-71-0o945-fig2.tif]
